# Statistical inference of chromosomal homology based on gene colinearity and applications to *Arabidopsis *and rice

**DOI:** 10.1186/1471-2105-7-447

**Published:** 2006-10-12

**Authors:** Xiyin Wang, Xiaoli Shi, Zhe Li, Qihui Zhu, Lei Kong, Wen Tang, Song Ge, Jingchu Luo

**Affiliations:** 1 College of Life Sciences, National Laboratory of Plant Genetic Engineering and Protein Engineering, Center of Bioinformatics, Peking University, Beijing 100871, China; 2 College of Mathematics, Hebei Polytechnic University, Tangshan, Hebei 063009, China; 3 Laboratory of Systematic and Evolutionary Botany, Institute of Botany, Chinese Academy of Sciences, Beijing 100093, China; 4 Beijing Genomics Institute, Chinese Academy of Sciences, Beijing 101300, China

## Abstract

**Background:**

The identification of chromosomal homology will shed light on such mysteries of genome evolution as DNA duplication, rearrangement and loss. Several approaches have been developed to detect chromosomal homology based on gene synteny or colinearity. However, the previously reported implementations lack statistical inferences which are essential to reveal actual homologies.

**Results:**

In this study, we present a statistical approach to detect homologous chromosomal segments based on gene colinearity. We implement this approach in a software package ColinearScan to detect putative colinear regions using a dynamic programming algorithm. Statistical models are proposed to estimate proper parameter values and evaluate the significance of putative homologous regions. Statistical inference, high computational efficiency and flexibility of input data type are three key features of our approach.

**Conclusion:**

We apply ColinearScan to the *Arabidopsis *and rice genomes to detect duplicated regions within each species and homologous fragments between these two species. We find many more homologous chromosomal segments in the rice genome than previously reported. We also find many small colinear segments between rice and Arabidopsis genomes.

## Background

Exploration of homology between chromosomes facilitates the understanding of the structure, function and evolution of genomes. Extensive synteny and colinearity have been detected between chromosomes in different species of cereals [1], mammals [2] and yeasts [3] providing a deep insight into the evolution of ancient chromosomes. Between chromosomes of the same species, large-scale homologous segments exist caused by whole genome or segmental duplication [4-9]. It has been reported that nearly 80% of the *Arabidopsis thaliana *genome and 45–60% of the rice genome are in large duplicated regions [10-12].

Special care should be taken to reveal chromosomal homology due to numerous genomic changes such as chromosomal rearrangements, gene inversions and gene loss [13-15]. Many approaches have been developed to identify chromosomal homologues [16] based on genetic maps [17], sequence alignment [18,19], gene synteny [10] and gene colinearity [20-23]. By detecting the density and order of homologous gene pairs between chromosomes, colinearity approach can reveal reliable homologous regions and requires less computational resources. This approach also enables us to develop reasonable statistical tests to evaluate the significance of predicted homologous regions.

The typical implementations of the colinearity strategy are ADHoRe [20], FISH [24] and DiagHunter [25]. The implementations of these approaches have limitations in some aspects, though they have been widely adopted. Firstly, the gap size between neighboring homologous genes which is essential to define and detect true colinearity needs further evaluation [12,20-23,26]. Secondly, statistical tests to assess predicted homologous regions are mainly based on a prerequisite of balanced gene loss rates between homologous regions. Finally, compositional and structural differences, especially gene density and repetition in genome-wide and local chromosomal regions, have not been fully addressed.

Here we describe a new colinearity approach characterized by improved statistical inference, flexibility and computational efficiency. Firstly, the selection of parameter values is theoretically explored, especially that of the gap length between neighboring genes. Secondly, the statistical test has been substantially strengthened with a mathematical deduction to evaluate the significance of the predicted homologous regions. Finally, the compositional and structural heterogeneity of chromosomes has been considered.

Using a dynamic programming algorithm, we developed ColinearScan and scanned the *Arabidopsis *and rice genomes to detect duplicated regions in each species and homologous chromosomal regions between these two species. We found 75.0% of *Arabidopsis *genes and 76.2% of rice genes were in duplicated regions. Moreover, we identified homologous fragments between these two species, in 32.9% of *Arabidopsis *and 16.8% of rice. Nearly all homologous segments were shorter than 0.6 Mb, indicating massive chromosomal rearrangements after the monocot-dicot divergence [27].

## Results

### Algorithm

#### Gene homology matrix

**Figure 1:**
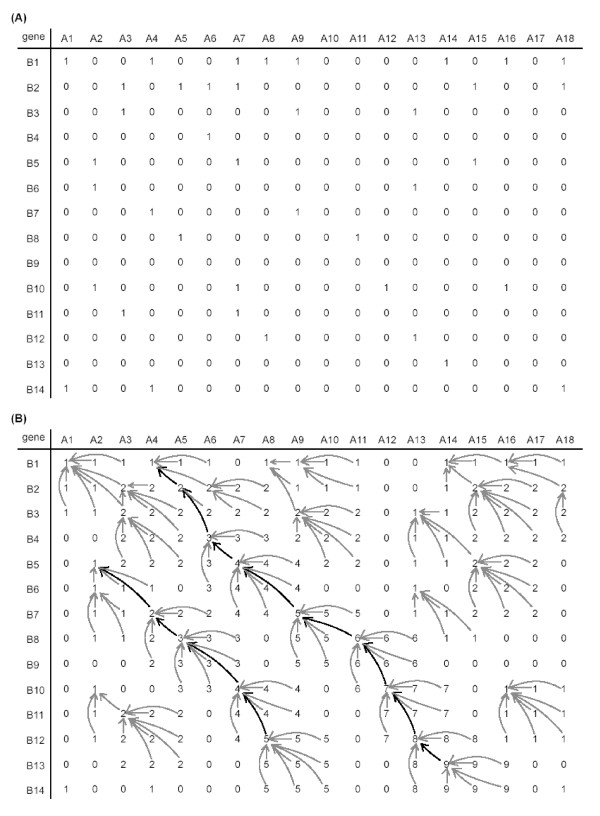
**A modified Smith-Waterman algorithm to locate colinearity**. (A) A simplified gene homology matrix (GHM, denoted as *H*). Genes A1, A2, ..., A18 on chromosome A are arranged horizontally, and genes B1, B2, ..., B14 on chromosome B are arranged vertically. Each cell of the matrix is filled with "1" or "0" based on the homology information from BLASTP search, e.g., gene A1 and gene B1 are homologous, and gene A2 and B2 are non-homologous. (B) A modified dynamic programming procedure. A scoring matrix *S *is constructed recursively based on *H*, with *mg *set to 2 genes apart. The distance criterion demands that neighboring genes in colinearity are no more than 2 genes apart. Pointers are shown by dark or grey arrow lines. Two collinear paths containing 9 and 5 genes are shown by dark arrow lines reflecting the same colinear relationship between the corresponding chromosomal regions.

**Figure 2:**
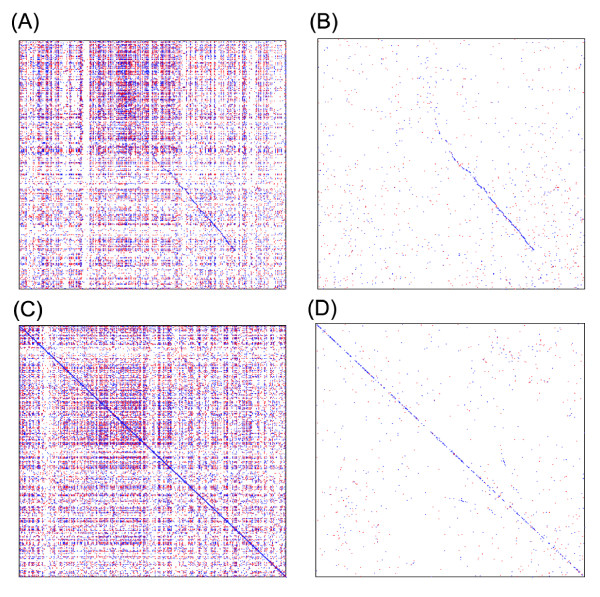
**Examples of dot maps**. (A) A dot map between rice chromosomes 2 and 4. Each dot in the map reflects a homologous gene pair with BLASTP score > 100. The dots are not distributed uniformly in the map. The map is also featured by many horizontal and vertical lines formed by repetitive genes. (B) A dot map between the same chromosomes as (A) with repetitive genes filtered. (C) A dot map of rice chromosome 1 against itself. Self-matching dots form a solid diagonal line. (D) A dot map with self-matching and repetitive genes filtered. A diagonal line reflecting the neighboring homologues can still be seen.

The first step in our colinearity approach is the construction of the gene homology matrix. To find homologous gene pairs between two chromosomes denoted as A and B, protein sequences encoded by genetically or physically positioned genes are used to perform an all-against-all BLAST search [28]. A gene homology matrix (GHM, denoted as *H*) is then constructed using the homology information from BLAST results. Chromosome A and B, represented by the positioned genes are arranged along *H *horizontally and vertically (Fig. 1A). A cell of *H *is filled with "1" if the corresponding genes on chromosome A and chromosome B are homologous, otherwise with "0". Tandem and other repetitive genes are widely distributed in chromosomes showing many "1"s in horizontal or vertical straight lines in the dot matrix map (Fig. 2) and causing problems in revealing true homology. Therefore, we used a general approach, masking the genes appearing more than 10 times in both chromosomes.

#### Dynamic programming algorithm

To reveal the homologous genes in colinearity between two chromosomes, we implemented a dynamic programming approach based on the well-known Smith-Waterman algorithm [29]. Using this approach, we can discover the longest putative sister regions represented by several proximal points of colinear homologous gene pairs in nearly diagonal orientations. The points may not be in close proximity due to large-scale gene loss, insertion and translocation. The extent of the proximity of the points is essential to reveal and evaluate the colinear sister segments. Lines forming by the points corresponding to the true colinear segments are either nearly parallel to the main diagonal line or the anti-diagonal line due to DNA segmental inversion. Homologous genes in colinear segments should all have the same or inverse transcriptional directions if no single gene inversions occur. We scan *H *in two directions, starting from the upper-left and upper-right. Here, we describe the procedure starting from the upper-left, which also applies in the other direction. Transcriptional orientations of the genes are recorded but not used when performing the colinearity search.

To reveal the colinearity represented by the proximal points in *H*, we introduce a parameter *mg *(the maximum gap length) between two neighboring points. Then we define another matrix *S *(the scoring matrix) with the same size as *H *(Fig. 1B). A cell in matrix *S *represents the extension of a colinearity path, i.e., the value of each cell is the number of collinear gene pairs in the path accumulated from its starting point. The path extends and the value of the cell increases by 1 if there is a "1" in lower-right neighborhood, and both vertical and horizontal distances are less than *mg*.

Initially, *S *is identical to *H*. We rebuild the matrix *S *recursively using a dynamic programming procedure:

S(i,j)=max{S(i-1,j-1)+H(i,j),if dist(p(i,j), p(i-1,j-1))<mg and S(i-1,j-1)>S(pre(i-1,j-1))S(pre(i-1,j-1))+H(i,j),if dist(p(i,j), pre(i-1,j-1)) <mg and  S(i-1,j-1)=S(pre(i-1,j-1))S(i-1,j),if dist(p(i,j), p(i-1,j))<mg and  S(i-1,j)>S(pre(i-1,j))S(pre(i-1,j)),if dist(p(i,j), pre(i-1,j))<mg and  S(i-1,j)=S(pre(i-1,j))S(i,j-1),if dist(p(i,j), p(i,j-1))<mg and S(i,j-1)>S(pre(i,j-1))S(pre(i,j-1)),if dist(p(i,j), pre(i,j-1))<mg and S(i,j-1)=S(pre(i,j-1))H(i,j),or else
 MathType@MTEF@5@5@+=feaafiart1ev1aaatCvAUfKttLearuWrP9MDH5MBPbIqV92AaeXatLxBI9gBaebbnrfifHhDYfgasaacH8akY=wiFfYdH8Gipec8Eeeu0xXdbba9frFj0=OqFfea0dXdd9vqai=hGuQ8kuc9pgc9s8qqaq=dirpe0xb9q8qiLsFr0=vr0=vr0dc8meaabaqaciaacaGaaeqabaqabeGadaaakeaacqqGtbWucqqGOaakcqqGPbqAcqqGSaalcqqGQbGAcqqGPaqkcqGH9aqpcqqGTbqBcqqGHbqycqqG4baEdaGabeqaauaabaqahiaaaaqaaiabbofatjabbIcaOiabbMgaPjabb2caTiabbgdaXiabbYcaSiabbQgaQjabb2caTiabbgdaXiabbMcaPiabgUcaRiabbIeaijabbIcaOiabbMgaPjabbYcaSiabbQgaQjabbMcaPiabbYcaSaqaaiabbMgaPjabbAgaMjabbccaGiabbsgaKjabbMgaPjabbohaZjabbsha0jabbIcaOiabbchaWjabbIcaOiabbMgaPjabbYcaSiabbQgaQjabbMcaPiabbYcaSiabbccaGiabbchaWjabbIcaOiabbMgaPjabb2caTiabbgdaXiabbYcaSiabbQgaQjabb2caTiabbgdaXiabbMcaPiabbMcaPiabgYda8iabb2gaTjabbEgaNjabbccaGiabbggaHjabb6gaUjabbsgaKjabbccaGiabbofatjabbIcaOiabbMgaPjabb2caTiabbgdaXiabbYcaSiabbQgaQjabb2caTiabbgdaXiabbMcaPiabg6da+iabbofatjabbIcaOiabbchaWjabbkhaYjabbwgaLjabbIcaOiabbMgaPjabb2caTiabbgdaXiabbYcaSiabbQgaQjabb2caTiabbgdaXiabbMcaPiabbMcaPaqaaiabbofatjabbIcaOiabbchaWjabbkhaYjabbwgaLjabbIcaOiabbMgaPjabb2caTiabbgdaXiabbYcaSiabbQgaQjabb2caTiabbgdaXiabbMcaPiabbMcaPiabgUcaRiabbIeaijabbIcaOiabbMgaPjabbYcaSiabbQgaQjabbMcaPiabbYcaSaqaaiabbMgaPjabbAgaMjabbccaGiabbsgaKjabbMgaPjabbohaZjabbsha0jabbIcaOiabbchaWjabbIcaOiabbMgaPjabbYcaSiabbQgaQjabbMcaPiabbYcaSiabbccaGiabbchaWjabbkhaYjabbwgaLjabbIcaOiabbMgaPjabb2caTiabbgdaXiabbYcaSiabbQgaQjabb2caTiabbgdaXiabbMcaPiabbMcaPiabbccaGiabgYda8iabb2gaTjabbEgaNjabbccaGiabbggaHjabb6gaUjabbsgaKjabbccaGiabbccaGiabbofatjabbIcaOiabbMgaPjabb2caTiabbgdaXiabbYcaSiabbQgaQjabb2caTiabbgdaXiabbMcaPiabg2da9iabbofatjabbIcaOiabbchaWjabbkhaYjabbwgaLjabbIcaOiabbMgaPjabb2caTiabbgdaXiabbYcaSiabbQgaQjabb2caTiabbgdaXiabbMcaPiabbMcaPaqaaiabbofatjabbIcaOiabbMgaPjabb2caTiabbgdaXiabbYcaSiabbQgaQjabbMcaPiabbYcaSaqaaiabbMgaPjabbAgaMjabbccaGiabbsgaKjabbMgaPjabbohaZjabbsha0jabbIcaOiabbchaWjabbIcaOiabbMgaPjabbYcaSiabbQgaQjabbMcaPiabbYcaSiabbccaGiabbchaWjabbIcaOiabbMgaPjabb2caTiabbgdaXiabbYcaSiabbQgaQjabbMcaPiabbMcaPiabgYda8iabb2gaTjabbEgaNjabbccaGiabbggaHjabb6gaUjabbsgaKjabbccaGiabbccaGiabbofatjabbIcaOiabbMgaPjabb2caTiabbgdaXiabbYcaSiabbQgaQjabbMcaPiabg6da+iabbofatjabbIcaOiabbchaWjabbkhaYjabbwgaLjabbIcaOiabbMgaPjabb2caTiabbgdaXiabbYcaSiabbQgaQjabbMcaPiabbMcaPaqaaiabbofatjabbIcaOiabbchaWjabbkhaYjabbwgaLjabbIcaOiabbMgaPjabb2caTiabbgdaXiabbYcaSiabbQgaQjabbMcaPiabbMcaPiabbYcaSaqaaiabbMgaPjabbAgaMjabbccaGiabbsgaKjabbMgaPjabbohaZjabbsha0jabbIcaOiabbchaWjabbIcaOiabbMgaPjabbYcaSiabbQgaQjabbMcaPiabbYcaSiabbccaGiabbchaWjabbkhaYjabbwgaLjabbIcaOiabbMgaPjabb2caTiabbgdaXiabbYcaSiabbQgaQjabbMcaPiabbMcaPiabgYda8iabb2gaTjabbEgaNjabbccaGiabbggaHjabb6gaUjabbsgaKjabbccaGiabbccaGiabbofatjabbIcaOiabbMgaPjabb2caTiabbgdaXiabbYcaSiabbQgaQjabbMcaPiabg2da9iabbofatjabbIcaOiabbchaWjabbkhaYjabbwgaLjabbIcaOiabbMgaPjabb2caTiabbgdaXiabbYcaSiabbQgaQjabbMcaPiabbMcaPaqaaiabbofatjabbIcaOiabbMgaPjabbYcaSiabbQgaQjabb2caTiabbgdaXiabbMcaPiabbYcaSaqaaiabbMgaPjabbAgaMjabbccaGiabbsgaKjabbMgaPjabbohaZjabbsha0jabbIcaOiabbchaWjabbIcaOiabbMgaPjabbYcaSiabbQgaQjabbMcaPiabbYcaSiabbccaGiabbchaWjabbIcaOiabbMgaPjabbYcaSiabbQgaQjabb2caTiabbgdaXiabbMcaPiabbMcaPiabgYda8iabb2gaTjabbEgaNjabbccaGiabbggaHjabb6gaUjabbsgaKjabbccaGiabbofatjabbIcaOiabbMgaPjabbYcaSiabbQgaQjabb2caTiabbgdaXiabbMcaPiabg6da+iabbofatjabbIcaOiabbchaWjabbkhaYjabbwgaLjabbIcaOiabbMgaPjabbYcaSiabbQgaQjabb2caTiabbgdaXiabbMcaPiabbMcaPaqaaiabbofatjabbIcaOiabbchaWjabbkhaYjabbwgaLjabbIcaOiabbMgaPjabbYcaSiabbQgaQjabb2caTiabbgdaXiabbMcaPiabbMcaPiabbYcaSaqaaiabbMgaPjabbAgaMjabbccaGiabbsgaKjabbMgaPjabbohaZjabbsha0jabbIcaOiabbchaWjabbIcaOiabbMgaPjabbYcaSiabbQgaQjabbMcaPiabbYcaSiabbccaGiabbchaWjabbkhaYjabbwgaLjabbIcaOiabbMgaPjabbYcaSiabbQgaQjabb2caTiabbgdaXiabbMcaPiabbMcaPiabgYda8iabb2gaTjabbEgaNjabbccaGiabbggaHjabb6gaUjabbsgaKjabbccaGiabbofatjabbIcaOiabbMgaPjabbYcaSiabbQgaQjabb2caTiabbgdaXiabbMcaPiabg2da9iabbofatjabbIcaOiabbchaWjabbkhaYjabbwgaLjabbIcaOiabbMgaPjabbYcaSiabbQgaQjabb2caTiabbgdaXiabbMcaPiabbMcaPaqaaiabbIeaijabbIcaOiabbMgaPjabbYcaSiabbQgaQjabbMcaPiabbYcaSaqaaiabb+gaVjabbkhaYjabbccaGiabbwgaLjabbYgaSjabbohaZjabbwgaLbaaaiaawUhaaaaa@21C9@

where *S*(*i*, *j*) is the score computed, *H*(*i*, *j*) is the homology information, *pre*(*i*, *j*) is the cell leading to the maximum score at the cell *p*(*i*, *j*), and a pointer (denoted by dark or gray arrow lines in Fig. 1B) is created from the cell *p*(*i*, *j*) to *pre*(*i*, *j*), *dist*(*p*(*i*, *j*), *p*(*a*, *b*)) is the distance between the cells *p*(*i*, *j*) and *p*(*a*, *b*). Eventually, the maximum score in *S *corresponds to the longest putative collinear segments. The longest colinearity path formed by dots of the homologous gene pairs is revealed by a trace-back procedure according to the pointers created. After the homologous genes in putative colinearity are recorded, we mask these putative colinear segments by setting *H*(*i*, *j*) to 0, rebuild the matrix *S*, and scan for other putative colinear segments till no sister regions containing more colinear genes than a threshold *r *could be found.

#### Maximum gap length

In colinearity methods, *mg *is the most important parameter which determines the length, quality and extensiveness of the predicated colinearity. The frequency of gene deletion in duplicated chromosomal segments is high and only a small fraction of homologous genes remains in colinearity. A small value of *mg *will result in finding many small colinear segments, and increase the difficulty of interpreting possible evolutionary events. On the other hand, a large *mg *value will surely result in high false positives. In fact, *mg *is dependent on the density of homologous gene pairs between the chromosomes. When the two chromosomes A and B are from the same species, homologous genes between them are mainly distributed at a similar density. On the other hand, when we compare two chromosomes from different species, the density of homologous genes may more divergent. Therefore we adopt two parameters to define the maximum distance between the neighboring dots, the maximum gap between genes in chromosome A (*mgA*) and the maximum gap between genes in chromosome B (*mgB*). When the chromosomes are from the same species, we set *mgA *= *mgB*.

Under the assumption that homologous genes are uniformly distributed in chromosomes, we explore the possibility of finding sister segments containing equal to or more than *r *genes by chance. However, this uniform distribution assumption is not very strict since we only need reasonable rather than optimal values of *mg*. Suppose the length of chromosome A and B are *lenA *and *lenB*, and the number of homologous gene pairs between two chromosomes is *pnum*. The location of a gene is a random variable with a probability density 1lenA
 MathType@MTEF@5@5@+=feaafiart1ev1aaatCvAUfKttLearuWrP9MDH5MBPbIqV92AaeXatLxBI9gBaebbnrfifHhDYfgasaacH8akY=wiFfYdH8Gipec8Eeeu0xXdbba9frFj0=OqFfea0dXdd9vqai=hGuQ8kuc9pgc9s8qqaq=dirpe0xb9q8qiLsFr0=vr0=vr0dc8meaabaqaciaacaGaaeqabaqabeGadaaakeaadaWcaaqaaiabigdaXaqaaiabdYgaSjabdwgaLjabd6gaUjabdgeabbaaaaa@32D0@, the joint probability density of the locations of *r *genes on chromosome A is 1[lenA]⥄r
 MathType@MTEF@5@5@+=feaafiart1ev1aaatCvAUfKttLearuWrP9MDH5MBPbIqV92AaeXatLxBI9gBaebbnrfifHhDYfgasaacH8akY=wiFfYdH8Gipec8Eeeu0xXdbba9frFj0=OqFfea0dXdd9vqai=hGuQ8kuc9pgc9s8qqaq=dirpe0xb9q8qiLsFr0=vr0=vr0dc8meaabaqaciaacaGaaeqabaqabeGadaaakeaadaWcaaqaaiabigdaXaqaamaadmaabaGaemiBaWMaemyzauMaemOBa4MaemyqaeeacaGLBbGaayzxaaWaaWbaaSqabeaacaaMcSUaemOCaihaaaaaaaa@37E3@, and the joint probability density of the locations on chromosome A and B of *r *homologous pairs is 1[lenA⋅lenB]⥄r
 MathType@MTEF@5@5@+=feaafiart1ev1aaatCvAUfKttLearuWrP9MDH5MBPbIqV92AaeXatLxBI9gBaebbnrfifHhDYfgasaacH8akY=wiFfYdH8Gipec8Eeeu0xXdbba9frFj0=OqFfea0dXdd9vqai=hGuQ8kuc9pgc9s8qqaq=dirpe0xb9q8qiLsFr0=vr0=vr0dc8meaabaqaciaacaGaaeqabaqabeGadaaakeaadaWcaaqaaiabigdaXaqaamaadmaabaGaemiBaWMaemyzauMaemOBa4MaemyqaeKaeyyXICTaemiBaWMaemyzauMaemOBa4MaemOqaieacaGLBbGaayzxaaWaaWbaaSqabeaacaaMcSUaemOCaihaaaaaaaa@3F53@. Therefore, the probability *p *that *r *homologous gene pairs are in colinearity by chance can be evaluated by

Ppnumr•[∫D1[lenA⋅lenB]rdx1dx2⋯dxrdy1dy2⋯dyr],
 MathType@MTEF@5@5@+=feaafiart1ev1aaatCvAUfKttLearuWrP9MDH5MBPbIqV92AaeXatLxBI9gBaebbnrfifHhDYfgasaacH8akY=wiFfYdH8Gipec8Eeeu0xXdbba9frFj0=OqFfea0dXdd9vqai=hGuQ8kuc9pgc9s8qqaq=dirpe0xb9q8qiLsFr0=vr0=vr0dc8meaabaqaciaacaGaaeqabaqabeGadaaakeaacqWGqbaudaqhaaWcbaGaemiCaaNaemOBa4MaemyDauNaemyBa0gabaGaemOCaihaaOGaeyOiGCRaei4waS1aa8quaeaadaWcaaqaaiabigdaXaqaaiabcUfaBjabdYgaSjabdwgaLjabd6gaUjabdgeabjabgwSixlabdYgaSjabdwgaLjabd6gaUjabdkeacjabc2faDnaaCaaaleqabaGaemOCaihaaaaaaeaacqWGebaraeqaniabgUIiYdGccqWGKbazcqWG4baEdaWgaaWcbaGaeGymaedabeaakiabdsgaKjabdIha4naaBaaaleaacqaIYaGmaeqaaOGaeS47IWKaemizaqMaemiEaG3aaSbaaSqaaiabdkhaYbqabaGccqWGKbazcqWG5bqEdaWgaaWcbaGaeGymaedabeaakiabdsgaKjabdMha5naaBaaaleaacqaIYaGmaeqaaOGaeS47IWKaemizaqMaemyEaK3aaSbaaSqaaiabdkhaYbqabaGccqGGDbqxcqGGSaalaaa@6B8A@

where the multiple integral field *D *is

[∩i=2r{xi|0<xi−xi−1<mgA⥄}]∩{xi|0<xi<lenA}∩[∩i=2r{yi|0<yi−yi−1<mgB⥄}]∩{yi|0<yi<lenB}
 MathType@MTEF@5@5@+=feaafiart1ev1aaatCvAUfKttLearuWrP9MDH5MBPbIqV92AaeXatLxBI9gBaebbnrfifHhDYfgasaacH8akY=wiFfYdH8Gipec8Eeeu0xXdbba9frFj0=OqFfea0dXdd9vqai=hGuQ8kuc9pgc9s8qqaq=dirpe0xb9q8qiLsFr0=vr0=vr0dc8meaabaqaciaacaGaaeqabaqabeGadaaakeaacqGGBbWwdaafWbqaaiabcUha7jabdIha4naaBaaaleaacqWGPbqAaeqaaOGaeiiFaWNaeGimaaJaeyipaWJaemiEaG3aaSbaaSqaaiabdMgaPbqabaGccqGHsislcqWG4baEdaWgaaWcbaGaemyAaKMaeyOeI0IaeGymaedabeaakiabgYda8iabd2gaTjabdEgaNjabdgeabjaaykW6cqGG9bqFcqGGDbqxaSqaaiabdMgaPjabg2da9iabikdaYaqaaiabdkhaYbqdcqWIPissaOGaeSykIKKaei4EaSNaemiEaG3aaSbaaSqaaiabdMgaPbqabaGccqGG8baFcqaIWaamcqGH8aapcqWG4baEdaWgaaWcbaGaemyAaKgabeaakiabgYda8iabdYgaSjabdwgaLjabd6gaUjabdgeabjabc2ha9jablMIijjabcUfaBnaauahabaGaei4EaSNaemyEaK3aaSbaaSqaaiabdMgaPbqabaGccqGG8baFcqaIWaamcqGH8aapcqWG5bqEdaWgaaWcbaGaemyAaKgabeaakiabgkHiTiabdMha5naaBaaaleaacqWGPbqAcqGHsislcqaIXaqmaeqaaOGaeyipaWJaemyBa0Maem4zaCMaemOqaiKaaGPaRlabc2ha9jabc2faDbWcbaGaemyAaKMaeyypa0JaeGOmaidabaGaemOCaihaniablMIijbGccqWIPisscqGG7bWEcqWG5bqEdaWgaaWcbaGaemyAaKgabeaakiabcYha8jabicdaWiabgYda8iabdMha5naaBaaaleaacqWGPbqAaeqaaOGaeyipaWJaemiBaWMaemyzauMaemOBa4MaemOqaiKaeiyFa0haaa@98FB@

and *x*_1_,*x*_2_,...,*x*_*r*_;*y*_1_,*y*_2_,...,*y*_*r *_are the positions of the genes on the chromosomes, Ppnumr
 MathType@MTEF@5@5@+=feaafiart1ev1aaatCvAUfKttLearuWrP9MDH5MBPbIqV92AaeXatLxBI9gBaebbnrfifHhDYfgasaacH8akY=wiFfYdH8Gipec8Eeeu0xXdbba9frFj0=OqFfea0dXdd9vqai=hGuQ8kuc9pgc9s8qqaq=dirpe0xb9q8qiLsFr0=vr0=vr0dc8meaabaqaciaacaGaaeqabaqabeGadaaakeaacqWGqbaudaqhaaWcbaGaemiCaaNaemOBa4MaemyDauNaemyBa0gabaGaemOCaihaaaaa@3513@ is the number of permutations of homologous genes: pnum!(pnum−r)!
 MathType@MTEF@5@5@+=feaafiart1ev1aaatCvAUfKttLearuWrP9MDH5MBPbIqV92AaeXatLxBI9gBaebbnrfifHhDYfgasaacH8akY=wiFfYdH8Gipec8Eeeu0xXdbba9frFj0=OqFfea0dXdd9vqai=hGuQ8kuc9pgc9s8qqaq=dirpe0xb9q8qiLsFr0=vr0=vr0dc8meaabaqaciaacaGaaeqabaqabeGadaaakeaadaWcaaqaaiabdchaWjabd6gaUjabdwha1jabd2gaTjabcgcaHaqaaiabcIcaOiabdchaWjabd6gaUjabdwha1jabd2gaTjabgkHiTiabdkhaYjabcMcaPiabcgcaHaaaaaa@3DA4@. When *mgA *<<*lenA *and *mgB *<<*lenB*, the integral in the above formula can be approximated by

1[lenA⋅lenB]⥄r(∫0lenA−mgAdx1∫x1x1+mgAdx2⋯∫xr−1xr−1+mgAdxr)(∫0lenB−mgBdy1∫y1y1+mgBdy2⋯∫yr−1yr−1+mgBdyr)
 MathType@MTEF@5@5@+=feaafiart1ev1aaatCvAUfKttLearuWrP9MDH5MBPbIqV92AaeXatLxBI9gBaebbnrfifHhDYfgasaacH8akY=wiFfYdH8Gipec8Eeeu0xXdbba9frFj0=OqFfea0dXdd9vqai=hGuQ8kuc9pgc9s8qqaq=dirpe0xb9q8qiLsFr0=vr0=vr0dc8meaabaqaciaacaGaaeqabaqabeGadaaakeaadaWcaaqaaiabigdaXaqaamaadmaabaGaemiBaWMaemyzauMaemOBa4MaemyqaeKaeyyXICTaemiBaWMaemyzauMaemOBa4MaemOqaieacaGLBbGaayzxaaWaaWbaaSqabeaacaaMcSUaemOCaihaaaaakiabcIcaOmaapedabaGaemizaqMaemiEaG3aaSbaaSqaaiabigdaXaqabaaabaGaeGimaadabaGaemiBaWMaemyzauMaemOBa4MaemyqaeKaeyOeI0IaemyBa0Maem4zaCMaemyqaeeaniabgUIiYdGcdaWdXaqaaiabdsgaKjabdIha4naaBaaaleaacqaIYaGmaeqaaaqaaiabdIha4naaBaaameaacqaIXaqmaeqaaaWcbaGaemiEaG3aaSbaaWqaaiabigdaXaqabaWccqGHRaWkcqWGTbqBcqWGNbWzcqWGbbqqa0Gaey4kIipakiabl+UimnaapedabaGaemizaqMaemiEaG3aaSbaaSqaaiabdkhaYbqabaGccqGGPaqkcqGGOaakdaWdXaqaaiabdsgaKjabdMha5naaBaaaleaacqaIXaqmaeqaaaqaaiabicdaWaqaaiabdYgaSjabdwgaLjabd6gaUjabdkeacjabgkHiTiabd2gaTjabdEgaNjabdkeacbqdcqGHRiI8aaWcbaGaemiEaG3aaSbaaWqaaiabdkhaYjabgkHiTiabigdaXaqabaaaleaacqWG4baEdaWgaaadbaGaemOCaiNaeyOeI0IaeGymaedabeaaliabgUcaRiabd2gaTjabdEgaNjabdgeabbqdcqGHRiI8aOWaa8qmaeaacqWGKbazcqWG5bqEdaWgaaWcbaGaeGOmaidabeaaaeaacqWG5bqEdaWgaaadbaGaeGymaedabeaaaSqaaiabdMha5naaBaaameaacqaIXaqmaeqaaSGaey4kaSIaemyBa0Maem4zaCMaemOqaieaniabgUIiYdGccqWIVlctdaWdXaqaaiabdsgaKjabdMha5naaBaaaleaacqWGYbGCaeqaaaqaaiabdMha5naaBaaameaacqWGYbGCcqGHsislcqaIXaqmaeqaaaWcbaGaemyEaK3aaSbaaWqaaiabdkhaYjabgkHiTiabigdaXaqabaWccqGHRaWkcqWGTbqBcqWGNbWzcqWGcbGqa0Gaey4kIipakiabcMcaPaaa@B2A4@

Then we can estimate *p *by

Ppnumr(mgA⋅mgBlenA⋅lenB)r−1.
 MathType@MTEF@5@5@+=feaafiart1ev1aaatCvAUfKttLearuWrP9MDH5MBPbIqV92AaeXatLxBI9gBaebbnrfifHhDYfgasaacH8akY=wiFfYdH8Gipec8Eeeu0xXdbba9frFj0=OqFfea0dXdd9vqai=hGuQ8kuc9pgc9s8qqaq=dirpe0xb9q8qiLsFr0=vr0=vr0dc8meaabaqaciaacaGaaeqabaqabeGadaaakeaacqWGqbaudaqhaaWcbaGaemiCaaNaemOBa4MaemyDauNaemyBa0gabaGaemOCaihaaOGaeiikaGYaaSaaaeaacqWGTbqBcqWGNbWzcqWGbbqqcqGHflY1cqWGTbqBcqWGNbWzcqWGcbGqaeaacqWGSbaBcqWGLbqzcqWGUbGBcqWGbbqqcqGHflY1cqWGSbaBcqWGLbqzcqWGUbGBcqWGcbGqaaGaeiykaKYaaWbaaSqabeaacqWGYbGCcqGHsislcqaIXaqmaaGccqGGUaGlaaa@51AE@

If we set the significance level of colinearity to α, then we have

Ppnumr(mgA⋅mgBlenA⋅lenB)r−1<α.
 MathType@MTEF@5@5@+=feaafiart1ev1aaatCvAUfKttLearuWrP9MDH5MBPbIqV92AaeXatLxBI9gBaebbnrfifHhDYfgasaacH8akY=wiFfYdH8Gipec8Eeeu0xXdbba9frFj0=OqFfea0dXdd9vqai=hGuQ8kuc9pgc9s8qqaq=dirpe0xb9q8qiLsFr0=vr0=vr0dc8meaabaqaciaacaGaaeqabaqabeGadaaakeaacqWGqbaudaqhaaWcbaGaemiCaaNaemOBa4MaemyDauNaemyBa0gabaGaemOCaihaaOGaeiikaGYaaSaaaeaacqWGTbqBcqWGNbWzcqWGbbqqcqGHflY1cqWGTbqBcqWGNbWzcqWGcbGqaeaacqWGSbaBcqWGLbqzcqWGUbGBcqWGbbqqcqGHflY1cqWGSbaBcqWGLbqzcqWGUbGBcqWGcbGqaaGaeiykaKYaaWbaaSqabeaacqWGYbGCcqGHsislcqaIXaqmaaGccqGH8aapiiGacqWFXoqycqGGUaGlaaa@5458@

When the two chromosomes are from the same species, we assume *mgA *= *mgB *and denote them as *mg*, and the value of *mg *can be evaluated by

mg<[(αPpnumr)1r−1(lenA⋅lenB)]12.
 MathType@MTEF@5@5@+=feaafiart1ev1aaatCvAUfKttLearuWrP9MDH5MBPbIqV92AaeXatLxBI9gBaebbnrfifHhDYfgasaacH8akY=wiFfYdH8Gipec8Eeeu0xXdbba9frFj0=OqFfea0dXdd9vqai=hGuQ8kuc9pgc9s8qqaq=dirpe0xb9q8qiLsFr0=vr0=vr0dc8meaabaqaciaacaGaaeqabaqabeGadaaakeaacqWGTbqBcqWGNbWzcqGH8aapdaWadaqaaiabcIcaOmaalaaabaacciGae8xSdegabaGaemiuaa1aa0baaSqaaiabdchaWjabd6gaUjabdwha1jabd2gaTbqaaiabdkhaYbaaaaGccqGGPaqkdaahaaWcbeqaamaalaaabaGaeGymaedabaGaemOCaiNaeyOeI0IaeGymaedaaaaakiabcIcaOiabdYgaSjabdwgaLjabd6gaUjabdgeabjabgwSixlabdYgaSjabdwgaLjabd6gaUjabdkeacjabcMcaPaGaay5waiaaw2faamaaCaaaleqabaWaaSaaaeaacqaIXaqmaeaacqaIYaGmaaaaaOGaeiOla4caaa@5409@

When the two chromosomes are from different species, the gene densities are often different and thus we adopt different values for the parameters *mgA *and *mgB*. The length of gaps between neighboring homologous genes is inversely proportional to the density of homologous genes, let

mgAmgB=pnumlenB⋅lenApnum=lenAlenB.
 MathType@MTEF@5@5@+=feaafiart1ev1aaatCvAUfKttLearuWrP9MDH5MBPbIqV92AaeXatLxBI9gBaebbnrfifHhDYfgasaacH8akY=wiFfYdH8Gipec8Eeeu0xXdbba9frFj0=OqFfea0dXdd9vqai=hGuQ8kuc9pgc9s8qqaq=dirpe0xb9q8qiLsFr0=vr0=vr0dc8meaabaqaciaacaGaaeqabaqabeGadaaakeaadaWcaaqaaiabd2gaTjabdEgaNjabdgeabbqaaiabd2gaTjabdEgaNjabdkeacbaacqGH9aqpdaWcaaqaaiabdchaWjabd6gaUjabdwha1jabd2gaTbqaaiabdYgaSjabdwgaLjabd6gaUjabdkeacbaacqGHflY1daWcaaqaaiabdYgaSjabdwgaLjabd6gaUjabdgeabbqaaiabdchaWjabd6gaUjabdwha1jabd2gaTbaacqGH9aqpdaWcaaqaaiabdYgaSjabdwgaLjabd6gaUjabdgeabbqaaiabdYgaSjabdwgaLjabd6gaUjabdkeacbaacqGGUaGlaaa@598E@

Thus, we can estimate the value of *mgA *and *mgB*

mgA<(αPpnumr)12(r−1)lenA,
 MathType@MTEF@5@5@+=feaafiart1ev1aaatCvAUfKttLearuWrP9MDH5MBPbIqV92AaeXatLxBI9gBaebbnrfifHhDYfgasaacH8akY=wiFfYdH8Gipec8Eeeu0xXdbba9frFj0=OqFfea0dXdd9vqai=hGuQ8kuc9pgc9s8qqaq=dirpe0xb9q8qiLsFr0=vr0=vr0dc8meaabaqaciaacaGaaeqabaqabeGadaaakeaacqWGTbqBcqWGNbWzcqWGbbqqcqGH8aapcqGGOaakdaWcaaqaaGGaciab=f7aHbqaaiabdcfaqnaaDaaaleaacqWGWbaCcqWGUbGBcqWG1bqDcqWGTbqBaeaacqWGYbGCaaaaaOGaeiykaKYaaWbaaSqabeaadaWcaaqaaiabigdaXaqaaiabikdaYiabcIcaOiabdkhaYjabgkHiTiabigdaXiabcMcaPaaaaaGccqWGSbaBcqWGLbqzcqWGUbGBcqWGbbqqcqGGSaalaaa@4A77@

mgB<(αPpnumr)12(r−1)lenB.
 MathType@MTEF@5@5@+=feaafiart1ev1aaatCvAUfKttLearuWrP9MDH5MBPbIqV92AaeXatLxBI9gBaebbnrfifHhDYfgasaacH8akY=wiFfYdH8Gipec8Eeeu0xXdbba9frFj0=OqFfea0dXdd9vqai=hGuQ8kuc9pgc9s8qqaq=dirpe0xb9q8qiLsFr0=vr0=vr0dc8meaabaqaciaacaGaaeqabaqabeGadaaakeaacqWGTbqBcqWGNbWzcqWGcbGqcqGH8aapcqGGOaakdaWcaaqaaGGaciab=f7aHbqaaiabdcfaqnaaDaaaleaacqWGWbaCcqWGUbGBcqWG1bqDcqWGTbqBaeaacqWGYbGCaaaaaOGaeiykaKYaaWbaaSqabeaadaWcaaqaaiabigdaXaqaaiabikdaYiabcIcaOiabdkhaYjabgkHiTiabigdaXiabcMcaPaaaaaGccqWGSbaBcqWGLbqzcqWGUbGBcqWGcbGqcqGGUaGlaaa@4A7F@

#### Colinearity shadow

**Figure 3:**
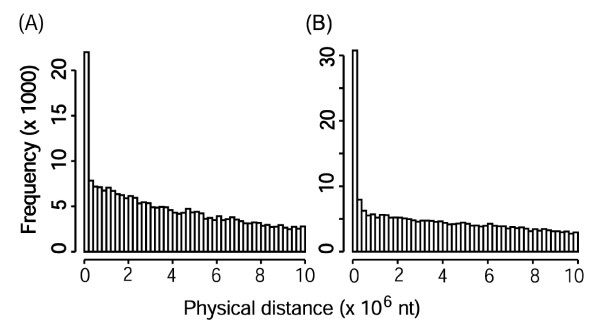
**Distance distribution of homologous genes**. (A) The distance distribution of rice homologous genes. (B) The distance distribution of *Arabidopsis *homologous genes.

Many genes have homologues in their neighborhood as indicated by the distance distribution of homologous genes (Fig. 3). Neighboring homologues may result in many colinear segments parallel to each other in sister regions within the same chromosome, or between the different chromosomes (Fig. 2A, 2C). If for convenience we call the longer ones the 'true' colinearity, then the shorter ones are the 'shadows'. The colinearity shadows may also reflect colinearity, but they do not provide further information and greatly increase the computational time. Thus after a putative colinearity in certain sister regions is found, we mask the neighboring homologous pairs within the corresponding the entire rectangular regions to avoid possible colinearity shadows.

When candidate colinearity is found in a specific region of GHM from upper-left to lower-right, colinear shadows perpendicular to this path might also be found in the same region scanning from upper-right to lower-left. These shadows may also reflect actual homology between two sister chromosomal segments, and they may occur when gene rearrangements are frequent in specific regions. A large amount of perpendicular shadows may affect efficiency of the algorithm. However, they do not occur very often so we do not mask these shadows.

### Statistical test

Many genes are in multi-gene families and repetitive genes are extensively found in plant genomes. Putative colinear regions might be a reflection of extensive occurrences of single gene duplication or translocation. Therefore, it is critical to correctly detect the colinearity pattern by evaluating the significance of the putative colinear regions generated by the above approach, and to develop an effective statistical method to assess whether the putative colinearity represents true homology or is produced by chance. Moreover, the distribution of homologous gene pairs is far from uniform. We use a statistical test considering divergent distribution of homologous gene pairs in different regions, rather than assuming a uniform distribution throughout the genome.

Given that a DNA segment resides in chromosome B, a corresponding colinear segment in chromosome A is generated due to many independent single-gene duplication events. Under the assumption of a uniform distribution of collinearly paired genes in the local chromosomal regions, we obtain the probability *epvA *to display the significance of the homology,

epvA=RA1⋅∏i=2mωAi⋅RAipssA,
 MathType@MTEF@5@5@+=feaafiart1ev1aaatCvAUfKttLearuWrP9MDH5MBPbIqV92AaeXatLxBI9gBaebbnrfifHhDYfgasaacH8akY=wiFfYdH8Gipec8Eeeu0xXdbba9frFj0=OqFfea0dXdd9vqai=hGuQ8kuc9pgc9s8qqaq=dirpe0xb9q8qiLsFr0=vr0=vr0dc8meaabaqaciaacaGaaeqabaqabeGadaaakeaacqWGLbqzcqWGWbaCcqWG2bGDcqWGbbqqcqGH9aqpcqWGsbGudaqhaaWcbaGaemyqaeeabaGaeGymaedaaOGaeyyXIC9aaebCaeaadaWcaaqaaGGaciab=L8a3naaDaaaleaacqWGbbqqaeaacqWGPbqAaaGccqGHflY1cqWGsbGudaqhaaWcbaGaemyqaeeabaGaemyAaKgaaaGcbaGaemiCaaNaem4CamNaem4CamNaemyqaeeaaaWcbaGaemyAaKMaeyypa0JaeGOmaidabaGaemyBa0ganiabg+GivdGccqGGSaalaaa@504F@

where *m *is the number of collinear gene pairs, ωAi
 MathType@MTEF@5@5@+=feaafiart1ev1aaatCvAUfKttLearuWrP9MDH5MBPbIqV92AaeXatLxBI9gBaebbnrfifHhDYfgasaacH8akY=wiFfYdH8Gipec8Eeeu0xXdbba9frFj0=OqFfea0dXdd9vqai=hGuQ8kuc9pgc9s8qqaq=dirpe0xb9q8qiLsFr0=vr0=vr0dc8meaabaqaciaacaGaaeqabaqabeGadaaakeaaiiGacqWFjpWDdaqhaaWcbaGaemyqaeeabaGaemyAaKgaaaaa@3113@ is the gap length between paired gene *i *and gene *i-1*, RAi
 MathType@MTEF@5@5@+=feaafiart1ev1aaatCvAUfKttLearuWrP9MDH5MBPbIqV92AaeXatLxBI9gBaebbnrfifHhDYfgasaacH8akY=wiFfYdH8Gipec8Eeeu0xXdbba9frFj0=OqFfea0dXdd9vqai=hGuQ8kuc9pgc9s8qqaq=dirpe0xb9q8qiLsFr0=vr0=vr0dc8meaabaqaciaacaGaaeqabaqabeGadaaakeaacqWGsbGudaqhaaWcbaGaemyqaeeabaGaemyAaKgaaaaa@306C@ is the number of occurrences of the *i*-th paired gene and its homologues in the putative sister segment in chromosome A, *pssA *is the length of the sister segment in chromosome A. The above formula can be deduced by extending the colinearity point by point taking repetitive homologues in consideration. The possibility of finding such colinearity under this assumption will be increased by RAi
 MathType@MTEF@5@5@+=feaafiart1ev1aaatCvAUfKttLearuWrP9MDH5MBPbIqV92AaeXatLxBI9gBaebbnrfifHhDYfgasaacH8akY=wiFfYdH8Gipec8Eeeu0xXdbba9frFj0=OqFfea0dXdd9vqai=hGuQ8kuc9pgc9s8qqaq=dirpe0xb9q8qiLsFr0=vr0=vr0dc8meaabaqaciaacaGaaeqabaqabeGadaaakeaacqWGsbGudaqhaaWcbaGaemyqaeeabaGaemyAaKgaaaaa@306C@ times if RAi
 MathType@MTEF@5@5@+=feaafiart1ev1aaatCvAUfKttLearuWrP9MDH5MBPbIqV92AaeXatLxBI9gBaebbnrfifHhDYfgasaacH8akY=wiFfYdH8Gipec8Eeeu0xXdbba9frFj0=OqFfea0dXdd9vqai=hGuQ8kuc9pgc9s8qqaq=dirpe0xb9q8qiLsFr0=vr0=vr0dc8meaabaqaciaacaGaaeqabaqabeGadaaakeaacqWGsbGudaqhaaWcbaGaemyqaeeabaGaemyAaKgaaaaa@306C@ homologous genes of the *i*-th colinear gene exist in this segment. Similarly, we can define the probability *epvB*

epvB=RB1⋅∏i=2mωBi⋅RBipssB.
 MathType@MTEF@5@5@+=feaafiart1ev1aaatCvAUfKttLearuWrP9MDH5MBPbIqV92AaeXatLxBI9gBaebbnrfifHhDYfgasaacH8akY=wiFfYdH8Gipec8Eeeu0xXdbba9frFj0=OqFfea0dXdd9vqai=hGuQ8kuc9pgc9s8qqaq=dirpe0xb9q8qiLsFr0=vr0=vr0dc8meaabaqaciaacaGaaeqabaqabeGadaaakeaacqWGLbqzcqWGWbaCcqWG2bGDcqWGcbGqcqGH9aqpcqWGsbGudaqhaaWcbaGaemOqaieabaGaeGymaedaaOGaeyyXIC9aaebCaeaadaWcaaqaaGGaciab=L8a3naaDaaaleaacqWGcbGqaeaacqWGPbqAaaGccqGHflY1cqWGsbGudaqhaaWcbaGaemOqaieabaGaemyAaKgaaaGcbaGaemiCaaNaem4CamNaem4CamNaemOqaieaaaWcbaGaemyAaKMaeyypa0JaeGOmaidabaGaemyBa0ganiabg+GivdGccqGGUaGlaaa@505D@

Then we define

*epv *= max(*epvA*,*epvB*)

to measure the possibility of the colinearity appearing by chance in the sister regions. If it is below a threshold, we take the putative colinearity as significant.

**Figure 4:**
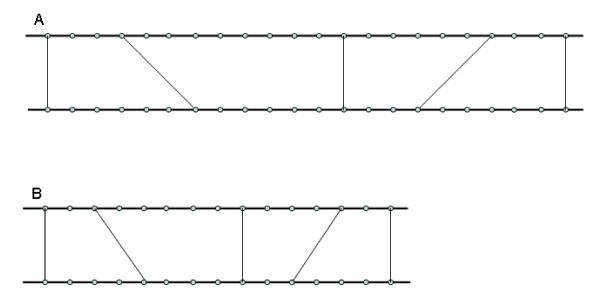
**Different distribution pattern of homologous genes in sister regions**. Same numbers of homologous genes located in two pairs of sister regions with different size. Homologue pairs are more densely located in (B) than in (A). Dark horizontal lines represent chromosomes, round dots denote genes on chromosomes, lines linking the dots indicate gene homology.

However, if we apply the above test directly to the putative different homologous regions, we cannot distinguish between patterns with similar numbers of homologous gene pairs in segments of different size. For example, the putative colinear regions found in small sister segments (Fig. 4B) more likely indicate true segmental homology than that in large sister segments (Fig. 4A). Dense colinearity possibly generated by recent duplications should be evaluated as significant.

Rather than using *pssA *and *pssB*, we try to estimate *epv *on expected scales of the sister regions (*essA *and *essB*) determined by the numbers of colinear genes and all homologous genes. Assuming that paired genes are uniformly distributed throughout the chromosomes, we estimate the expected sizes for each pair of sister segments as follows

essA=λ⋅cnumpnum⋅lenA,
 MathType@MTEF@5@5@+=feaafiart1ev1aaatCvAUfKttLearuWrP9MDH5MBPbIqV92AaeXatLxBI9gBaebbnrfifHhDYfgasaacH8akY=wiFfYdH8Gipec8Eeeu0xXdbba9frFj0=OqFfea0dXdd9vqai=hGuQ8kuc9pgc9s8qqaq=dirpe0xb9q8qiLsFr0=vr0=vr0dc8meaabaqaciaacaGaaeqabaqabeGadaaakeaacqWGLbqzcqWGZbWCcqWGZbWCcqWGbbqqcqGH9aqpiiGacqWF7oaBcqGHflY1daWcaaqaaiabdogaJjabd6gaUjabdwha1jabd2gaTbqaaiabdchaWjabd6gaUjabdwha1jabd2gaTbaacqGHflY1cqWGSbaBcqWGLbqzcqWGUbGBcqWGbbqqcqGGSaalaaa@4A7F@

essB=λ⋅cnumpnum⋅lenB,
 MathType@MTEF@5@5@+=feaafiart1ev1aaatCvAUfKttLearuWrP9MDH5MBPbIqV92AaeXatLxBI9gBaebbnrfifHhDYfgasaacH8akY=wiFfYdH8Gipec8Eeeu0xXdbba9frFj0=OqFfea0dXdd9vqai=hGuQ8kuc9pgc9s8qqaq=dirpe0xb9q8qiLsFr0=vr0=vr0dc8meaabaqaciaacaGaaeqabaqabeGadaaakeaacqWGLbqzcqWGZbWCcqWGZbWCcqWGcbGqcqGH9aqpiiGacqWF7oaBcqGHflY1daWcaaqaaiabdogaJjabd6gaUjabdwha1jabd2gaTbqaaiabdchaWjabd6gaUjabdwha1jabd2gaTbaacqGHflY1cqWGSbaBcqWGLbqzcqWGUbGBcqWGcbGqcqGGSaalaaa@4A83@

where *cnum *is the number of colinearly paired genes in the putative sister segments, *pnum *is the number of all homologous gene pairs between chromosome A and chromosome B, λ is a coefficient to normalize the size of different putative colinear segments. For each pair of putative sister regions we calculate a preliminary coefficient number λ_*i *_by (pssA⋅pnumlenA⋅cnum+pssB⋅pnumlenB⋅cnum)⋅12.
 MathType@MTEF@5@5@+=feaafiart1ev1aaatCvAUfKttLearuWrP9MDH5MBPbIqV92AaeXatLxBI9gBaebbnrfifHhDYfgasaacH8akY=wiFfYdH8Gipec8Eeeu0xXdbba9frFj0=OqFfea0dXdd9vqai=hGuQ8kuc9pgc9s8qqaq=dirpe0xb9q8qiLsFr0=vr0=vr0dc8meaabaqaciaacaGaaeqabaqabeGadaaakeaacqGGOaakdaWcaaqaaiabdchaWjabdohaZjabdohaZjabdgeabjabgwSixlabdchaWjabd6gaUjabdwha1jabd2gaTbqaaiabdYgaSjabdwgaLjabd6gaUjabdgeabjabgwSixlabdogaJjabd6gaUjabdwha1jabd2gaTbaacqGHRaWkdaWcaaqaaiabdchaWjabdohaZjabdohaZjabdkeacjabgwSixlabdchaWjabd6gaUjabdwha1jabd2gaTbqaaiabdYgaSjabdwgaLjabd6gaUjabdkeacjabgwSixlabdogaJjabd6gaUjabdwha1jabd2gaTbaacqGGPaqkcqGHflY1daWcaaqaaiabigdaXaqaaiabikdaYaaacqGGUaGlaaa@68F4@.

where *pssA *and *pssB *are the original sizes of the putative *i-th *sister segments. These preliminary coefficients are averaged throughout the putative homologous sister pairs to obtain the estimate of λ. Finally, we redefine epvA=RA1⋅∏i=2mωAi⋅RAiessA
 MathType@MTEF@5@5@+=feaafiart1ev1aaatCvAUfKttLearuWrP9MDH5MBPbIqV92AaeXatLxBI9gBaebbnrfifHhDYfgasaacH8akY=wiFfYdH8Gipec8Eeeu0xXdbba9frFj0=OqFfea0dXdd9vqai=hGuQ8kuc9pgc9s8qqaq=dirpe0xb9q8qiLsFr0=vr0=vr0dc8meaabaqaciaacaGaaeqabaqabeGadaaakeaacqWGLbqzcqWGWbaCcqWG2bGDcqWGbbqqcqGH9aqpcqWGsbGudaqhaaWcbaGaemyqaeeabaGaeGymaedaaOGaeyyXIC9aaebCaeaadaWcaaqaaGGaciab=L8a3naaDaaaleaacqWGbbqqaeaacqWGPbqAaaGccqGHflY1cqWGsbGudaqhaaWcbaGaemyqaeeabaGaemyAaKgaaaGcbaGaemyzauMaem4CamNaem4CamNaemyqaeeaaaWcbaGaemyAaKMaeyypa0JaeGOmaidabaGaemyBa0ganiabg+Givdaaaa@4F4F@ and epvB=RB1⋅∏i=2mωBi⋅RBiessB
 MathType@MTEF@5@5@+=feaafiart1ev1aaatCvAUfKttLearuWrP9MDH5MBPbIqV92AaeXatLxBI9gBaebbnrfifHhDYfgasaacH8akY=wiFfYdH8Gipec8Eeeu0xXdbba9frFj0=OqFfea0dXdd9vqai=hGuQ8kuc9pgc9s8qqaq=dirpe0xb9q8qiLsFr0=vr0=vr0dc8meaabaqaciaacaGaaeqabaqabeGadaaakeaacqWGLbqzcqWGWbaCcqWG2bGDcqWGcbGqcqGH9aqpcqWGsbGudaqhaaWcbaGaemOqaieabaGaeGymaedaaOGaeyyXIC9aaebCaeaadaWcaaqaaGGaciab=L8a3naaDaaaleaacqWGcbGqaeaacqWGPbqAaaGccqGHflY1cqWGsbGudaqhaaWcbaGaemOqaieabaGaemyAaKgaaaGcbaGaemyzauMaem4CamNaem4CamNaemOqaieaaaWcbaGaemyAaKMaeyypa0JaeGOmaidabaGaemyBa0ganiabg+Givdaaaa@4F59@

by substituting the original size *pssA *and *pssB *with the expected size *essA *and *essB*. Thus, we assign different significance to the two patterns in Fig. 4, the sister regions with denser collinear genes have a smaller *epv *value.

### Assessment of *mg *estimation

To test the applicability of the criteria defining *mg*, we performed a computational simulation test on rice chromosome 1 and *Arabidopsis *chromosome 1.

First, using the integral formula (*r *= 4, α = 0.01, length of rice chromosome 1: *lenA *= 48.2 Mb, length of *Arabidopsis *chromosome 1: *lenB *= 30.5 Mb, *pnum *= 1737), we calculated the maximum gap length in both chromosomes: *mgA *= 155 Kb, and *mgB *= 98 Kb. The parameters *r *and α mean that using such gap length to scan the homologous segments between the chromosomes, the probability of finding sister segments containing 4 or more collinear genes should be <= 0.01 under the assumption of uniform distribution.

Second, we shuffled the positions of the homologous gene pairs on both chromosomes and scanned the longest homologous segments occurring by chance, then checked whether it had 4 or more collinear genes. We repeated this process 1000 times and found collinear segment with 4 or more collinear genes 9 times.

We calculated *mgA *and *mgB *between every pair of chromosomes of rice and *Arabidopsis*, and applied the largest values (*mgA *= 160 Kb and *mgB *= 154 Kb) to all chromosome pairs. The candidate homologous segments can be verified by a statistical test. To check how it affects the scanning process between rice chromosome 1 and *Arabidopsis *chromosome 1 when using larger *mgA *and *mgB*, we found colinear segments with 4 or more collinear gene pairs in 33, out of 1000, simulations.

### Application to rice and *Arabidopsis*

We explored the colinear segments within and between the chromosomes in *Arabidopsis *and rice. The genomic sequences of *Arabidopsis thaliana *were from GenBank (Accession NC_003070, NC_003071, NC_003074, NC_003075, NC_003076) [30]. *Oryza sativa *L. *ssp. indica *genomic sequences were downloaded from RiceGD [31] and the rice genes were predicted using the software BGF [32] from the Beijing Genome Institute. By performing all-against-all BLASTP, we revealed homologous gene pairs within and between *Arabidopsis *and rice (BLASTP score > 100).

For *Arabidopsis*, we searched for the duplication regions with the parameter value *r *= 4, *mg *= 116 Kb (~25 intervening genes) and λ = 2.6. We used the maximal *mg *values estimated between each pairs of Arabidopsis chromosomes. At the significant level of *epv *<= 0.01, we discovered 203 duplicated sister segments out of 350 candidates, among them 3 were possible perpendicular shadows (Supplementary table 1 [see Additional file 1]). About 75.0% of the genome is in duplicated segments and 22.4%, 1.8% of the genes are in segments with a multiplication level > 2 and > 4, respectively (Table 1). The detected coverage of duplicated regions is a little more than that (71%) found by Blanc *et al*. [18], less than that (89%) reported by Bowers *et al*. [33]. The longest sister segments contain 106 colinear genes and extend more than 1.8 Mb in chromosome 2 and 1.55 Mb in chromosome 3 (Table 2). In the longest 20 duplicated segments, 88–100% of the colinear genes are of the same relative transcriptional orientation, indicating a low inversion rate.

**Table 1:** The number and percentage of genes in duplicated blocks in rice and Arabidopsis genomes

Multiplication level ^1^		2	3	4	5	6	7	8
Arabidopsis	Gene No	13452	3934	1345	269	115	86	0
	Percentage ^2^	0.750	0.224	0.071	0.018	0.008	0.003	0.000
Rice	Gene No	17947	11114	6020	2930	1371	846	550
	Percentage	0.762	0.429	0.223	0.111	0.057	0.031	0.016

^1^. Multiplication level of a gene displays that it is in a duplicated segment that appears for how many times in a genome.

^2^. The percentage is the ratio of genes in multiplication levels >= a specific number.

**Table 2:** The 20 longest duplicated segments in the *Arabidopsis *genome

Colinear gene number	Gene orientation identity	Epv	Segment A in *Arabidopsis*				Segment B in *Arabidopsis*			
				
			Starting gene	Ending gene	Gene number	Length (Mb)	Starting gene	Ending gene	Gene number	Length (Mb)
106	0.96	1.92E-226	2_3518	2_4078	560	1.88	3_4725	3_5165	440	1.55
83	0.96	3.99E-200	1_1726	1_2130	404	1.54	1_6013	1_6467	454	1.65
82	0.98	3.64E-199	1_6041	1_6467	426	1.56	1_1752	1_2130	378	1.45
78	0.97	1.83E-155	3_840	3_1141	301	0.97	5_151	5_609	458	1.54
70	0.90	1.38E-138	2_3108	2_3445	337	1.26	3_4285	3_4619	334	1.19
69	0.97	1.68E-129	1_3629	1_3991	362	1.43	3_1669	3_2096	427	1.56
64	0.94	1.23E-116	1_639	1_343	296	1.02	2_2172	2_2582	410	1.47
64	0.95	5.65E-127	3_118	3_353	235	0.75	5_1391	5_1691	300	1.05
62	1.00	2.82E-123	1_4258	1_4017	241	0.90	3_1328	3_1603	275	0.97
60	0.97	1.67E-114	4_1800	4_1590	210	0.78	5_3789	5_4037	248	0.90
59	0.97	1.24E-110	2_1607	2_1840	233	0.97	4_2963	4_3236	273	1.02
54	0.91	9.58E-101	4_2685	4_2484	201	0.68	5_4682	5_5062	380	1.35
53	0.98	6.29E-098	2_1326	2_1096	230	0.86	4_2727	4_2960	233	0.85
53	0.96	3.97E-091	3_2507	3_2114	393	1.57	4_1114	4_1412	298	1.27
51	0.94	6.88E-100	2_935	2_1093	158	0.62	4_3464	4_3625	161	0.57
41	0.98	1.07E-063	1_1423	1_1285	138	0.47	2_6	2_245	239	0.98
37	0.92	3.83E-062	2_1308	2_1472	164	0.56	4_3786	4_3958	172	0.57
37	0.89	1.33E-056	4_2327	4_2478	151	0.50	5_4245	5_4549	304	1.10
33	0.88	1.91E-044	1_134	1_271	137	0.44	4_191	4_396	205	0.83
32	1.00	6.68E-055	3_111	3_1	110	0.32	5_1250	5_1390	140	0.47

* The gene names (also in Table 4 and 5) reflect the chromosome and the gene order, e.g. '2_3518' represents the 3518th gene on chromosome 2.

As for rice, we used another set of parameter values *r *= 4, *mg *= 334 Kb (~46 intervening genes) and λ = 3.97. At the same significance level as in *Arabidopsis*, we revealed 309 duplicated segments out of 841 candidates in rice, among them 13 are possible perpendicular shadows (Supplementary table 2 [see Additional file 2]). In our study we found that 76.2% of the genes were in duplicated regions, significantly higher than 20.59% reported by Simillion *et al*. [20], also higher than those reported by Paterson *et al*. [10] (61.9%) and Guyot and Keller [5] (52%). The longest colinear sister regions contain 194 homologous genes and extend more than 4.11 and 3.73 Mb in chromosomes 11 and 12 (Table 3), corresponding a segmental duplication event duplicated ~5–7 Mya [15,34]. About 42.9%, 11.1% of the genome sequences are in a multiplication level of >2 and >4, respectively (Table 1). The transcriptional orientation of colinear genes is in high consistency, similar to that in *Arabidopsis*. The colinear sister segments at different levels of multiplication are distributed throughout the rice genome.

**Table 3:** The 20 longest duplicated segments in the rice genome

Colinear gene number	Gene orientation identity	Epv	Segment A in rice				Segment B in rice			
				
			Starting gene	Ending gene	Gene number	Length (Mb)	Starting gene	Ending gene	Gene number	Length (Mb)
194	0	0.95	11_5	11_700	695	4.11	12_73	12_691	618	3.73
191	0	0.95	2_3549	2_4505	956	6.18	4_3002	4_4139	1137	7.03
157	0	0.94	1_5264	1_3976	1288	8.42	5_3767	5_4423	656	3.85
139	0	0.97	1_6225	1_5317	908	5.68	5_2981	5_3719	738	4.50
126	3.65E-296	0.90	8_3168	8_4097	929	5.92	9_1944	9_2863	919	5.73
122	8.23E-286	0.89	3_2952	3_1610	1342	9.01	7_3177	7_4010	833	5.10
110	3.85E-257	0.94	2_5335	2_4716	619	3.94	6_682	6_1503	821	5.59
88	5.33E-191	0.95	2_2758	2_3516	758	5.07	4_2121	4_2941	820	5.56
59	2.57E-112	0.83	3_5355	3_5625	270	1.61	7_335	7_910	575	3.87
41	1.81E-067	0.98	1_743	1_1160	417	2.97	5_701	5_1067	366	2.75
40	4.49E-072	0.95	2_896	2_661	235	1.54	6_3717	6_4079	362	2.57
34	4.97E-054	0.97	3_3955	3_4295	340	2.28	12_2686	12_2997	311	2.08
33	1.83E-059	0.94	2_643	2_310	333	2.22	6_4127	6_4386	259	1.64
32	1.30E-051	0.94	2_1059	2_907	152	1.05	6_3325	6_3648	323	2.39
30	8.41E-049	0.90	2_1361	2_1112	249	1.76	6_2904	6_3209	305	2.18
23	1.49E-036	0.96	3_661	3_561	100	0.59	10_1721	10_1879	158	1.16
22	5.24E-030	1.00	1_6600	1_6816	216	1.30	5_2616	5_2780	164	1.15
22	9.83E-031	0.95	8_2936	8_3126	190	1.40	9_1646	9_1894	248	1.80
21	3.50E-038	0.95	1_581	1_724	143	0.74	5_559	5_644	85	0.56
21	3.23E-030	0.81	3_5157	3_5293	136	0.92	7_71	7_279	208	1.32

Using parameter values *r *= 4, *mgA *= 154 Kb in *Arabidopsis*, *mgB *= 160 Kb in rice and λ = 1.47, we found 177 colinear sister segments out of 432 candidates between the chromosomes of two species (Supplementary table 3 [see Additional file 3]), accounting for 32.9% and 16.9% of the *Arabidopsis *and rice genes, respectively. The longest sister segments are ~0.6 Mb in length and contain ~14 colinear genes, but most segments are much shorter, indicating extensive independent chromosomal rearrangements and gene loss or gain in each genome. The sister copy in rice is always 1–4 times longer than that in *Arabidopsis*, implying a possible chromosomal expansion in the rice genome (Table 4).

**Table 4:** The 20 longest collinear regions between the *Arabidopsis *and rice genome

Colinear gene number	Gene orientation identity	epv	Segment in *Arabidopsis*				Segments in rice			
				
			Starting gene	Ending gene	Gene number	Length (Mb)	Starting gene	Ending gene	Gene number	Length (Mb)

14	0.93	5.53E-16	4_3648	4_3741	93	0.61	2_3736	2_3815	79	0.32
11	0.64	1.45E-09	2_5222	2_5287	65	0.38	4_2954	4_3026	72	0.26
11	0.91	2.69E-10	4_2502	4_2430	72	0.46	2_2835	2_2883	48	0.19
10	0.60	1.37E-07	7_562	7_658	96	0.64	3_2215	3_2280	65	0.24
10	0.70	9.51E-08	10_3103	10_3021	82	0.54	2_3733	2_3781	48	0.19
10	0.70	3.61E-09	1_5674	1_5595	79	0.51	2_3286	2_3339	53	0.18
10	0.90	4.37E-10	8_3510	8_3590	80	0.42	5_4866	5_4931	65	0.23
9	0.78	7.81E-07	3_1660	3_1736	76	0.48	2_2221	2_2248	27	0.12
9	0.89	3.63E-07	2_4713	2_4635	78	0.41	5_4870	5_4948	78	0.29
9	0.56	4.60E-08	9_2767	9_2822	55	0.31	4_3435	4_3468	33	0.12
9	0.89	1.44E-08	7_3347	7_3305	42	0.28	1_5880	1_5908	28	0.10
9	1.00	4.51E-10	1_5843	1_5889	46	0.31	3_4249	3_4279	30	0.10
8	0.75	1.32E-05	5_3294	5_3386	92	0.56	5_104	5_198	94	0.29
8	0.75	6.45E-05	6_1100	6_1031	69	0.54	5_5811	5_5863	52	0.21
8	0.75	6.08E-05	7_3242	7_3189	53	0.32	5_5408	5_5441	33	0.12
8	0.75	4.70E-05	4_2502	4_2430	72	0.46	3_4109	3_4154	45	0.16
8	0.75	3.60E-05	4_3665	4_3741	76	0.50	3_4887	3_4952	65	0.23
8	0.63	2.24E-05	6_772	6_682	90	0.57	4_3081	4_3144	63	0.21
8	0.63	1.25E-05	4_2498	4_2425	73	0.49	2_1946	2_2009	63	0.19
8	0.63	1.12E-05	1_5796	1_5720	76	0.43	5_128	5_207	79	0.25

## Discussion

Identification of the duplicated segments, especially their distribution pattern in a genome, is essential for further inference on when and how the duplication or species divergence occurred, and whether or not recurrent duplication events happened. The selection of parameter values, in particular the maximum gap length between the neighboring genes, is critical to detect chromosomal homology. However, the selection of maximum gap length in previous reported studies was mainly empirical, which might fail to detect authentic duplicated segments [20,22,23]. Many fewer and shorter duplicated segments are discovered when a smaller gap length is adopted, such as in the case of rice [20], whereas more and longer duplicated regions can be found if a larger gap length is adopted. Moreover, different gap length should be used in different genomes such as *Arabidopsis *and rice, since the density of colinear genes varies due to DNA loss and insertion. By considering the difference in gene density, especially the density of homologous genes in different genomes, we determined the maximum gap length based on statistical analysis. For example, when the duplicated regions in *Arabidopsis *and rice are detected, the maximum gap lengths were estimated to be 116 Kb and 334 Kb, respectively.

The input data of our approach can be any type of genetic markers such as sequences, genetic markers. Various measurements can be used to represent the distance between markers, such as physical or genetic distances, or gene numbers. In most previous studies, the significance of the predicted colinear regions was evaluated by a permutation test, which is rather time-consuming [20,23]. We estimate the significance of the predicted colinear segments through statistical inference. The statistical inference has the advantage over the permutation test in terms of computational efficiency. It takes only 2 minutes to calculate the *epv *to evaluate their significance on a personal computer (AMD AthlonXP 2000+, 512 MB RAM) while running a permutation test takes several hours on the same machine. The massive gene duplications and translocations in its proximal regions will lead to many colinearity shadows, decreasing the computational efficiency. We include a neighborhood masking procedure in ColinearScan to remove colinearity shadows in our algorithm, which dramatically improves the efficiency of detecting duplicated segments in the rice genome.

ADHoRe adopts linear regression analysis to infer duplicated chromosomal segments [20,21]. The underlying assumption is that gene loss rates have been balanced between sister segments, resulting in a straight line in the dot map. The colinear homologues in a chromosomal segment might be interspersed by individual genes that have no homologues at the corresponding position in its sister segment. At the very beginning of divergence of the sister segments, there should be one-to-one gene homology. Thereafter, massive gene deletions, translocations and chromosomal rearrangements occur and the initial pattern eventually becomes obscured [25]. The homologues with the conservative orders would appear in a straight line in the dot map if gene deletion or insertion had been balanced in different regions of the sister segments, otherwise in a curvy line. Wang *et al*. [15] explore the gene loss rates in the sister segments in rice and find that nearly straight lines are obtained for some sister segments, *e.g*., in chromosomes 11 and 12, and in chromosomes 2 and 4. However, curvy lines are also found for some sister segments, *e.g*., in chromosomes 1 and 5, and in chromosomes 8 and 9. A linearity assumption might fail to detect true duplicated segments. In FISH, Calabrese *et al*. [24] also adopt a colinearity strategy and develop a different statistical approach to evaluate the extension of collinear points, referred as clump in GHM. However, the value of key parameter *p*, reflecting the probability that a point occurs in the neighborhood of the former point, is artificially defined, and the maximal gap is deduced from *p *in their approach. DiagHunter [25] adopts a colinearity method similar to our approach, and the maximal length of the path is predefined. The program stops extending the current path until it reaches the maximal length threshold, or other neighboring points cannot be found.

Polyploidy has been supposed to be prevalent in plants. Recently, genome-wide studies further suggest the ubiquity of polyploidy, even in genomes which have not been considered to undergo genomic duplication [35]. The small genome of *Arabidopsis *has been reported to have undergone at least one round of duplication by different groups [12,18]. Here using a different method, we discover that 75.0% of the *Arabidopsis *genome sequences are in duplicated regions and a significant portion of sequences have multiple copies. The previous studies in the rice genome have been focused on the large obvious duplicated segments, produced by the relatively recent duplication events [15,36]. Here, we detect 76.2% of rice sequences in duplicated regions, and 42.9% have multiple copies.

The possibility of constructing the monocot-dicot comparative genetic map has been discussed [37] based on the comparison of *Arabidopsis *and rice sequences [38,39]. However, a comprehensive detection of homologous regions between these two genomes has not been available. Based on gene colinearity, we detected homologous regions between *Arabidopsis *and rice, accounting for 32.9% and 16.9% of each genome. All homologous segments were shorter than 0.6 Mb in length, indicating the massive genome rearrangements in both genomes after the monocot-dicot divergence. Though the short homologous segments make it difficult to construct the comparative genetic map between monocot and dicot, the homologues in colinearity found in this study may provide clues for further work in comparative genomics.

## Conclusion

We develop an algorithm to detect homologous chromosomal segments with conserved gene order, and we propose a statistical approach to estimate parameters and evaluate the significance of potential homology. We apply this approach to rice and *Arabidopsis *with high efficiency to detect potential colinear regions and evaluate their significance. We find many more homologous chromosomal segments in rice genomes than previously reported, which consolidated the inference that a polyploidy had occurred in the common ancestor of grasses. We also find many small colinear segments between rice and *Arabidopsis *genomes, providing clues to the evolutionary history of monocots and dicots.

## Authors' contributions

XW and XS developed the algorithm and the statistical models under the supervision of JL. XW, XS and ZL implemented the programs in rice and *Arabidopsis*, QZ and SG contributed to this work on plant biology and evolution, ZL and LK developed the online web server, WT provided technical support to the project. All the authors contributed to the refinement of the manuscript drafted by XW.

## Availability and requirements

• Project name: ColinearScan

• Project home page: 

• Operating systems: Linux, Unix

• Programming languages: C++, PERL

• Other requirements: Standard C++ Library, BioPerl and other PERL modules including Getopt::Long and Pod::Usage

• License: GPL

## Supplementary Material

Additional file 1 Colinear blocks in Arabidopsis. A list of all collinear blocks in Arabidopsis Click here for file

Additional file 2 Colinear blocks in rice. A list of all collinear blocks in rice Click here for file

Additional file 3 Colinear blocks between rice and Arabidopsis. A list of all collinear blocks between rice and Arabidopsis Click here for file
